# Elite Marathoners Run Faster With Increasing Temperatures in Berlin Marathon

**DOI:** 10.3389/fphys.2021.649898

**Published:** 2021-07-07

**Authors:** Beat Knechtle, David Valero, Elias Villiger, José Ramón Alvero Cruz, Volker Scheer, Thomas Rosemann, Pantelis T. Nikolaidis

**Affiliations:** ^1^Medbase St. Gallen Am Vadianplatz, St. Gallen, Switzerland; ^2^Institute of Primary Care, University Hospital Zurich, Zurich, Switzerland; ^3^Ultra Sports Science Foundation, Pierre-Benite, France; ^4^Deparamento de Fisiología Humana, Histología, Anatomia Patológica y Educación Física y Deportiva, Málaga, Spain; ^5^School of Health and Caring Sciences, University of West Attica, Athens, Greece; ^6^Laboratory of Exercise Testing, Hellenic Air Force Academy, Acharnes, Greece

**Keywords:** running, heat, cold, rain, perfomance

## Abstract

The influence of environmental conditions has been investigated for different marathon races, but not for the Berlin Marathon, the fastest marathon race course in the world. The aim of this study was to investigate the potential influence of environmental conditions such as temperature, precipitation, sunshine, and atmospheric pressure on marathon race times in the Berlin Marathon since its first event in 1974–2019. A total of *n* = 882,540 valid finisher records were available for analysis, of which 724,135 correspond to male and 158,405 to female runners. We performed analyses regarding performance levels considering all finishers, the top 3, the top 10, and the top 100 women and men. Within the 46 years of Berlin marathons under study, there was some level of precipitation for 18 years, and 28 years without any rain. Sunshine was predominant in 25 of the events, whilst in the other 21, cloud cover was predominant. There was no significant trend with time in any of the weather variables (e.g., no increase in temperature across the years). Overall runners became slower with increasing temperature and sunshine duration, however, elite runners (i.e., top 3 and top 10) seemed to run faster and improved their race times when the temperature increased (with women improving more than men). Top 10 women seemed to benefit more from increasing temperatures than top 10 males, and male top 100 runners seemed to benefit more from increasing temperatures than female top 100 runners. In the top three sub-group, no differences were observed between male and female correlations. In summary, in marathoners competing in the Berlin Marathon between 1974 and 2019, increasing temperatures and sunshine duration showed a different effect on different performance levels where overall runners (i.e., the general mass of runners) became slower with increasing temperature and sunshine duration, but elite runners (i.e., top 3, top 10) became faster with increasing temperatures where sex differences exist.

## Introduction

It is well-known that environmental conditions such as ambient temperature (Cheuvront and Haymes, 2001; Nikolaidis et al., 2019), wind (Miller-Rushing et al., 2012), cloud cover (Trapasso and Cooper, 1989; Ely et al., 2007a), barometric pressure (Knechtle et al., 2019; Nikolaidis et al., 2019), and precipitation (Trapasso and Cooper, 1989; Knechtle et al., 2019; Nikolaidis et al., 2019) have an effect on marathon running performance. Indeed, an analysis investigating marathon race times of the World Marathon Major races for Boston, London, Berlin, Chicago, and New York showed that weather, rather than course, had an effect on race times (Maffetone et al., 2017).

Of all the weather variables, ambient temperature seems to have the highest influence on marathon race times (Zhang et al., 1992; Ely et al., 2007b). There is a lot of evidence that performance in a marathon is impaired with increasing temperature (Trapasso and Cooper, 1989; Ely et al., 2007b; González-Alonso, 2007; El Helou et al., 2012). The optimum temperature for a fast marathon race time is generally ∼10–12°C (Ely et al., 2007b; Maughan, 2010) or even as low as ∼8°C (Trapasso and Cooper, 1989).

The influence of temperature on marathon performance seems, however, to depend on the performance level (Ely et al., 2008; El Helou et al., 2012) where the optimum temperature for a fast marathon race time may be lower for faster runners than for slower runners (Maughan, 2010).

In some investigations, higher temperatures seemed to slow down faster runners compared to slower runners (Ely et al., 2008), whereas in other circumstances, slower runners suffered a greater performance decline than faster runners (Montain et al., 2007; Vihma, 2010).

The influence of ambient environmental conditions has been investigated for different marathon races such as the Boston Marathon (Trapasso and Cooper, 1989; Ely et al., 2007b; Miller-Rushing et al., 2012; Knechtle et al., 2019; Nikolaidis et al., 2019), the New York City Marathon (Ely et al., 2007b), the Stockholm Marathon (Vihma, 2010), and the Beijing Marathon (Zhang et al., 1992), but no study has yet investigated the influence of weather on marathon performance in all occurrences of the Berlin Marathon with the fastest race course and where most of the marathon world record times were set.

The aim of the present study was to investigate the influence of environmental conditions such as temperature (i.e., mean temperature, daily highest, and lowest temperature), sunshine duration, precipitation, barometric pressure on marathon race times in all editions of the Berlin Marathon since its first event in 1974 until 2019. Our hypothesis, based upon the existing findings for other marathon races, was to find an association between ambient temperatures and marathon race times where increasing temperatures would slow down faster runners more than slower runners.

## Materials and Methods

### Ethical Approval

This study was approved by the Institutional Review Board of Kanton St. Gallen, Switzerland, with a waiver of the requirement for informed consent of the participants as the study involved the analysis of publicly available data.

### Data Set and Data Preparation

The athlete data (name, surname, year of birth, sex, nationality) were obtained directly from the website of the Berlin Marathon^1^. We were able to download the entire dataset for each available year in JSON format and then convert it to an Excel file using a custom Python script. The weather data on race day was downloaded from the website of ‘‘Deutscher Wetterdienst’’^2^ with temperature (maximum, minimum, average in degrees Celsius), sunshine (duration in hours), precipitation (mm), cloud cover (duration in hours), and atmospheric pressure (mbar) and filtered for the respective race dates. We chose the data from the weather station Berlin Dahlem because of its proximity to the Berlin Marathon route.

### Data Processing

Two data files were used in this study: the first was a register of each Berlin marathon runner’s finishing times between 1974 and 2019 (with the exception of 1978 and 1980 for which no data was available), including the runner’s finish time in the format HH:MM:SS, along with their sex and age, and the year of the marathon. Since this study focused on the influence of weather conditions on finish times by performance tiers, the age information was not used. The second file was a register of the weather conditions during each of the marathons between 1974 and 2019, including temperature values (average, maximum, and minimum), and average atmospheric pressure and precipitation (along with other variables such as sunshine and cloud cover hours that are not used due to their high correlation with the temperatures). These files were visually inspected in an Excel spreadsheet first, where minor changes were made (renaming of header columns and removing of unused columns) and then uploaded into a Google Colab notebook, where Python was used to conduct the statistical processing and to create the results tables and charts. Given the main goal of performing descriptive statistics on the available data, the decision was made not to establish cut-off finish times on either end of the range.

### Statistical Analysis

Descriptive statistical analyses were performed on four nested performance tiers: top 3 finishers (elite), top 10, top 100, and all finishers, for males and females separately. Finish times of these performance groups were further filtered individually by each of the five originally continuous weather variables under consideration, converted into categories (ranges), as follows: Temperatures (degrees Celsius) are grouped in three ranges: 0–8, 8–15, and 15–30°C; atmospheric pressure values (mbar) in two ranges: 900–1013, 1013–1030 mbar; and precipitation values (mm) in three ranges: 0–10, 10–20, and 20–50 mm. These categories are selected based on existing results from the Boston Marathon (Knechtle et al., 2019; Nikolaidis et al., 2019). The resulting values of the marathon finish times are presented in terms of their average value (mean) and standard deviation (SD), along with maximum (max), and minimum (min) values for each category. The column named as “*n*” represents the number of samples in that specific category. The Kolmogorov–Smirnov two-sample test was applied to the male/female sub-populations to validate the assumption of the statistical significance of the resulting finishing times by gender. For the correlation analysis, we used both Pearson and Spearman correlation coefficients, given the varying degrees of normality of the variables compared and the top 3, top 10, and top 100 sampling process. We also explored the statistical significance of the correlations by calculating their associated *p*-values. The low coefficients obtained indicate weak correlations in general. However, this analysis was particularly insightful to identify differences in the effect of the weather conditions between males and females, and between runners in different performance levels. Statistical significance was set at 5% (*p* < 0.05) in all cases. All analyses were carried out using the Python programming language (Python Software Foundation^3^), in a Google Colab notebook^4^ and the Statistical Software for the Social Sciences (IBM SPSS v26, Chicago, IL, United States).

## Results

**FIGURE 1:**
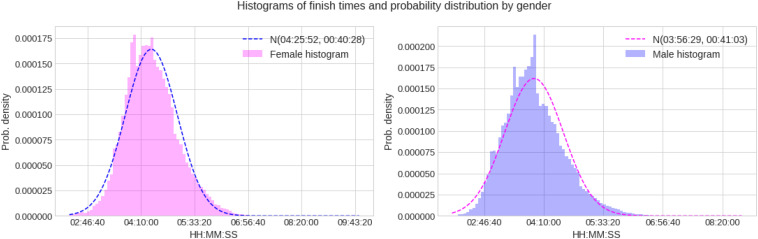
Marathon race times histograms and probability distributions for women and men.

After cleaning up and processing the data, a total of 882,540 valid finisher records were available for the analysis, of which 724,135 correspond to male runners and 158,405 to females. We performed analyses regarding performance levels considering all finishers, the top 3, the top 10, and the top 100 women and men. Figure 1 shows the distribution of marathon finish times for each gender.

### Weather Variables and Trends Across Years

**FIGURE 2:**
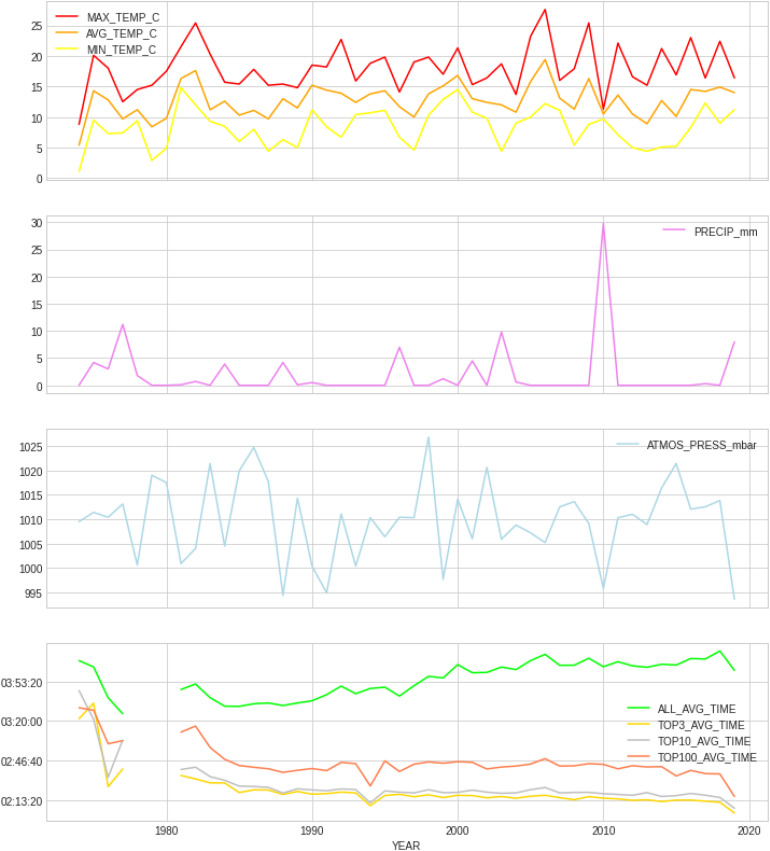
Average finish times by performance group (all, top 3, top 10, and top 100) along with profiles of measured weather variables from 1974 to 2019. PRECIP, precipitation in mm; ATMOS_PRESS, atmospheric pressure in mbar; AVG_TEMP, average temperature in degrees Celsius; MAX_TEMP, maximum temperature in degrees Celsius; MN_TEMP, minimum temperature in degrees Celsius.

Figure 2 presents the time profiles of the measured weather variables between 1974 and 2019, along with the average finish times of the four performance groups (all runners, top 3, top 10, and top 100) and Table 1 shows details of the weather variables with mean, SD, minimum, and maximum values. Within the 46 years of Berlin marathons under investigation, there was some level of precipitation in 18 of the years, while 28 years had no rain; sunshine was predominant in 25 of the events, whilst in the other 21 cloud cover was predominant. There was no significant trend with time in any of the weather variables (e.g., no increase in temperature across years).

**TABLE 1:** Details of the weather variables with mean, standard deviation (SD), minimum and maximum values (and percentiles); mm (millimeter), hrs. (hours), mbar (millibar), C (degrees Celsius).

	**Average temperature (°C)**	**Max temperature (°C)**	**Min temperature (°C)**	**Precipitation (mm)**	**Atmospheric pressure (mbar)**	**Sunshine (h)**	**Cloud (h)**
Mean	12.70	18.02	8.33	1.98	1009.80	5.63	4.49
SD	2.65	3.80	3.10	5.01	8.02	4.53	2.67
Min	5.40	8.80	1.10	0.00	993.62	0.00	0.10
25%	10.88	15.40	5.55	0.00	1005.38	0.70	2.00
50%	12.75	17.65	8.65	0.00	1010.35	6.95	5.00
75%	14.30	20.25	10.62	1.07	1014.03	10.05	7.00
Max	19.40	27.60	14.80	29.80	1026.80	11.40	8.00

### All Women and Men

**FIGURE 3:**
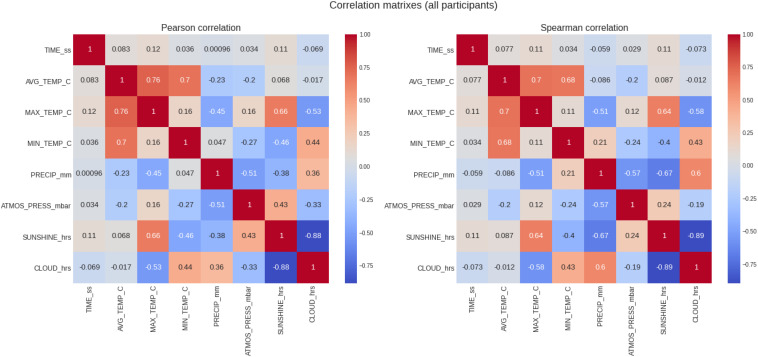
Analysis of the correlation of the target variable (marathon race time) and the descriptive variables (weather conditions) for all runners. PRECIP, precipitation in mm, SUNSHINE duration in hours, CLOUD cover in hours; ATMOS_PRESS, atmospheric pressure in mbar; AVG_TEMP, average temperature in degrees Celsius; MAX_TEMP, maximum temperature in degrees Celsius; MN_TEMP, minimum temperature in degrees Celsius.

Table 2 summarizes the results of the analysis for all runners and Figure 3 show the correlations (Pearson and Spearman), for the full sample without distinction of gender. Considering the correlation of marathon race time and the weather conditions, marathon race time showed a very weak correlation with all the weather variables, being only minimally significant with maximum temperature (*r* = 0.12, *p* = 0.0) and sunshine duration (*r* = 0.11, *p* = 0.0) where these two variables strongly correlated with each other (Pearson correlation, Figure 3). The difference in the correlation coefficients between males and females for the full sample (all runners) was negligible.

**TABLE 2:** All runners marathon finishing times in relation to ambient conditions; mm (millimeter), mbar (millibar), C (degrees Celsius), SD (standard deviation).

**Marathon finish time (all runners)**						
	**Gender**	***n***	**Mean**	**SD**	**Min**	**Max**
**Average temperature range (°C)**						
0–8	Female	10	04:40:46	00:38:15	03:22:01	05:40:10
	Male	234	04:09:36	00:47:49	02:44:53	05:55:53
8–15	Female	131,146	04:25:26	00:40:50	02:18:11	09:49:41
	Male	587,332	03:55:31	00:41:10	02:01:39	08:47:19
15–30	Female	27,249	04:27:59	00:38:38	02:19:12	07:00:54
	Male	136,569	04:00:34	00:40:17	02:05:56	07:58:43
**Maximum temperature range (°C)**						
8–15	Female	15,862	04:20:55	00:38:12	02:19:41	07:09:24
	Male	75,903	03:51:03	00:38:16	02:05:08	07:55:55
15–30	Female	14,2543	04:26:25	00:40:41	02:18:11	09:49:41
	Male	648,232	03:57:07	00:41:19	02:01:39	08:47:19
**Minimum temperature range (°C)**						
0–8	Female	62,829	04:23:00	00:39:37	02:19:19	07:07:05
	Male	276,800	03:52:59	00:40:24	02:02:57	07:55:55
8–15	Female	95,576	04:27:45	00:40:55	02:18:11	09:49:41
	Male	44,7335	03:58:38	00:41:18	02:01:39	08:47:19
**Atmospheric pressure range (mbar)**						
900–1013	Female	107,941	04:25:39	00:39:36	02:19:12	07:18:23
	Male	510,810	03:56:56	00:40:39	02:01:41	08:47:19
1013–1030	Female	50,464	04:26:19	00:42:18	02:18:11	09:49:41
	Male	213,325	03:55:22	00:41:57	02:01:39	08:30:02
**Precipitation range (mm)**						
0–10	Female	150,996	04:25:46	00:40:34	02:18:11	09:49:41
	Male	697,314	03:56:22	00:41:08	02:01:39	08:47:19
10–20	Female	12	03:52:22	00:40:34	02:40:38	04:54:20
	Male	217	03:24:43	00:32:47	02:16:20	05:17:56
20–50	Female	7397	04:27:49	00:38:26	02:23:58	07:09:24
	Male	26,604	03:59:38	00:38:38	02:05:08	07:07:24

### Top Three Women and Men

**FIGURE 4:**
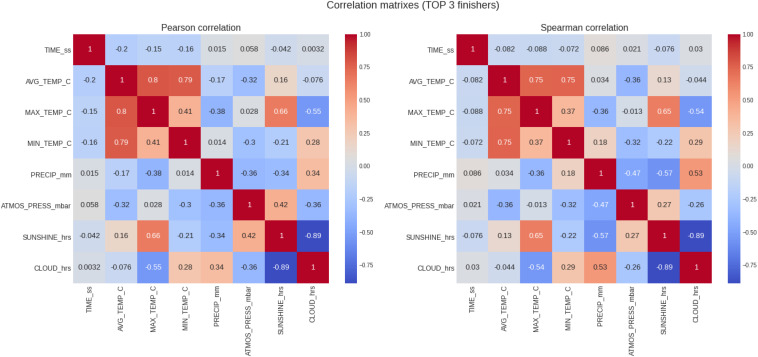
Analysis of the correlation of the target variable (marathon race time) and the descriptive variables (weather conditions) for the top three runners. PRECIP, precipitation in mm, SUNSHINE duration in hours, CLOUD cover in hours; ATMOS_PRESS, atmospheric pressure in mbar; AVG_TEMP, average temperature in degrees Celsius; MAX_TEMP, maximum temperature in degrees Celsius; MN_TEMP, minimum temperature in degrees Celsius.

The results of the analysis for the top three category and the correlations can be seen in Table 3 and Figure 4, respectively. In this group, a total of 255 runners with 132 males and 123 females were considered. The correlation between marathon race times and maximum temperature changes from positive to negative values for the elite runners (top three) in respect to the generic analysis (all runners). In other words, when the temperature increased, elite runners seemed to run faster and improve their race times whilst the masses (all runners) seemed to run slower and worsen their finish times. Correlation coefficients continued very low (*r* = −0.2, −0.15 for Pearson correlation of finish times with average and max temperatures, with *p* < 0.05) and Spearman correlation indicated and even weaker correlation. There was no significant difference in the correlation coefficients when the correlation analysis was performed separately for males and females.

**TABLE 3:** Top 3 marathon finishing times in relation to ambient conditions; mm (millimeter), mbar (millibar), C (degrees Celsius), SD (standard deviation).

**Marathon finish time (top 3 runners)**						
	**Gender**	***n***	**Mean**	**SD**	**Min**	**Max**
**Average temperature range (°C)**						
0–8	Female	3	03:57:28	00:32:44	03:22:01	04:26:35
	Male	3	02:46:34	00:01:37	02:44:53	02:48:08
8–15	Female	96	02:33:42	00:24:13	02:18:11	04:39:24
	Male	105	02:10:18	00:08:28	02:01:39	02:50:02
15–30	Female	24	02:31:51	00:10:57	02:19:12	02:53:56
	Male	24	02:10:12	00:03:55	02:05:56	02:20:10
**Maximum temperature range (°C)**						
8–15	Female	18	02:47:41	00:37:10	02:19:41	04:26:35
	Male	18	02:16:04	00:14:36	02:05:08	02:48:08
15–30	Female	105	02:32:52	00:22:56	02:18:11	04:39:24
	Male	114	02:10:19	00:08:09	02:01:39	02:50:02
**Minimum temperature range (°C)**						
0–8	Female	54	02:36:40	00:27:06	02:19:19	04:26:35
	Male	57	02:12:19	00:10:17	02:02:57	02:48:08
8–15	Female	69	02:33:46	00:24:58	02:18:11	04:39:24
	Male	75	02:10:10	00:08:41	02:01:39	02:50:02
**Atmospheric pressure range (mbar)**						
900–1013	Female	81	02:35:24	00:28:41	02:19:12	04:39:24
	Male	90	02:11:30	00:10:42	02:01:41	02:50:02
1013–1030	Female	42	02:34:19	00:19:35	02:18:11	03:41:31
	Male	42	02:10:14	00:05:54	02:01:39	02:30:07
**Precipitation range (mm)**						
0–10	Female	117	02:34:36	00:26:00	02:18:11	04:39:24
	Male	126	02:11:05	00:09:34	02:01:39	02:50:02
10–20	Female	3	03:01:56	00:18:34	02:40:38	03:14:46
	Male	3	02:17:30	00:01:28	02:16:20	02:19:10
20–50	Female	3	02:25:02	00:01:06	02:23:58	02:26:10
	Male	3	02:05:14	00:00:09	02:05:08	02:05:25

### Top 10 Women and Men

**FIGURE 5:**
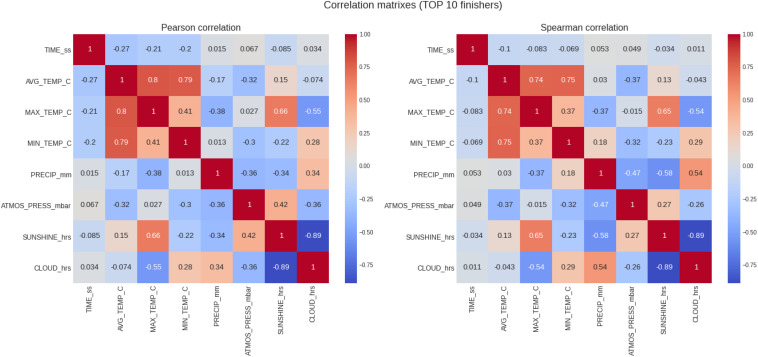
Analysis of the correlation of the target variable (marathon race time) and the descriptive variables (weather conditions) for the top 10 runners. PRECIP, precipitation in mm, SUNSHINE duration in hours, CLOUD cover in hours; ATMOS_PRESS, atmospheric pressure in mbar; AVG_TEMP, average temperature in degrees Celsius; MAX_TEMP, maximum temperature in degrees Celsius; MN_TEMP, minimum temperature in degrees Celsius.

Table 4 summarizes the results of the analysis for the top 10 runners and Figure 5 shows the correlations. In the top 10 were 844 records in total, with 440 of males and 404 of females. Marathon race times showed a slightly higher (but still weak) negative correlation with the temperature variables (i.e., average, minimum, and maximum temperature), similar to the results obtained for the top three runners. In this case, we can also observe a growing difference between the correlation coefficients for males and females. This would suggest a larger positive impact to female finish times than to males. For the top 10 women, the Pearson correlation coefficients were for average, maximum, and minimum temperature −0.37, −0.3, and −0.27, respectively, but for men −0.22, −0.17, and −0.2, respectively (*p* < 0.05 in all cases).

**TABLE 4:** Top 10 marathon finishing times in relation to ambient conditions; mm (millimeter), mbar (millibar), C (degrees Celsius), SD (standard deviation).

**Marathon finish time (top 10 runners)**						
	**Gender**	***n***	**Mean**	**SD**	**Min**	**Max**
**Average temperature range (°C)**						
0–8	Female	10	04:40:46	00:38:15	03:22:01	05:40:10
	Male	10	02:50:39	00:03:53	02:44:53	02:56:05
8–15	Female	314	02:37:44	00:24:03	02:18:11	04:56:30
	Male	350	02:13:30	00:09:40	02:01:39	02:58:55
15–30	Female	80	02:38:32	00:13:40	02:19:12	03:12:14
	Male	80	02:13:41	00:05:17	02:05:56	02:25:13
**Maximum temperature range (°C)**						
8–15	Female	60	03:04:06	00:54:38	02:19:41	05:40:10
	Male	60	02:19:45	00:15:44	02:05:08	02:56:05
15–30	Female	344	02:36:54	00:20:22	02:18:11	04:56:30
	Male	380	02:13:31	00:09:09	02:01:39	02:58:55
**Minimum temperature range (°C)**						
0–8	Female	180	02:45:58	00:38:43	02:19:19	05:40:10
	Male	190	02:15:54	00:11:37	02:02:57	02:56:05
8–15	Female	224	02:36:53	00:18:56	02:18:11	04:56:30
	Male	250	02:13:13	00:09:26	02:01:39	02:58:55
**Atmospheric pressure range (mbar)**						
900–1013	Female	264	02:39:52	00:30:31	02:19:12	05:40:10
	Male	300	02:14:40	00:11:36	02:01:41	02:58:55
1013–1030	Female	140	02:42:57	00:28:12	02:18:11	04:32:47
	Male	140	02:13:45	00:07:36	02:01:39	02:39:38
**Precipitation range (mm)**						
0–10	Female	384	02:39:40	00:28:20	02:18:11	05:40:10
	Male	420	02:14:13	00:10:28	02:01:39	02:58:55
10–20	Female	10	03:41:08	00:34:07	02:40:38	04:32:47
	Male	10	02:27:00	00:07:43	02:16:20	02:34:58
20–50	Female	10	02:29:22	00:03:54	02:23:58	02:34:47
	Male	10	02:08:31	00:02:44	02:05:08	02:12:42

### Top 100 Women and Men

**FIGURE 6:**
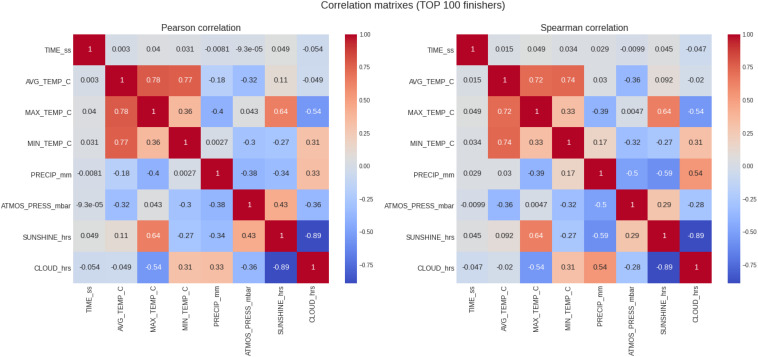
Analysis of the correlation of the target variable (marathon race time) and the descriptive variables (weather conditions) for the top 100 runners. PRECIP, precipitation in mm; SUNSHINE duration in hours, CLOUD cover in hours; ATMOS_PRESS, atmospheric pressure in mbar; AVG_TEMP, average temperature in degrees Celsius; MAX_TEMP, maximum temperature in degrees Celsius; MN_TEMP, minimum temperature in degrees Celsius.

The results of the analysis for the top 100 runners and the correlations can be seen in Table 5 and Figure 6, respectively. In the top 100 were 8141 records with 4400 males and 3741 females. The correlation coefficients (Figure 6) for the top 100 runners (males and females combined) dropped to negligible levels (both in their Pearson and Spearman modalities), and the statistical significance test (Kolmogorov–Smirnov test) threw a *p*-value of 1.0 (meaning a we cannot discard the top 100 male and female sub-samples proceed from the same probability distribution), which is an anomaly. However, these coefficients showed an interesting result when calculated separately for males and females. For the top 100 women, the Pearson correlation coefficients were for average, maximum, and minimum temperature 0.14, 0.16, and 0.21, but for men −0.21, −0.14, and −0.17 (*p* < 0.05 in all cases), respectively. Male top 100 runners seemed to benefit more than female top 100 runners from increasing temperatures. Positive correlation coefficients for females indicate worsening times with increasing temperatures. Negative coefficients for males indicate rather the opposite effect, that is, better times with increasing temperatures.

**TABLE 5:** Top 100 marathon finishing times in relation to ambient conditions; mm (millimeter), mbar (millibar), C (degrees Celsius), SD (standard deviation).

**Marathon finish time (top 100 runners)**						
	**Gender**	***n***	**Mean**	**SD**	**Min**	**Max**
**Average temperature range (°C)**						
0–8	Female	10	04:40:46	00:38:15	03:22:01	05:40:10
	Male	100	03:24:17	00:18:20	02:44:53	03:52:44
8–15	Female	2931	03:00:13	00:16:44	02:18:11	04:56:30
	Male	3500	02:28:42	00:16:34	02:01:39	03:51:37
15–30	Female	800	03:13:51	00:27:02	02:19:12	04:36:26
	Male	800	02:29:31	00:09:31	02:05:56	02:46:59
**Maximum temperature range (°C)**						
8–15	Female	422	03:01:24	00:23:24	02:19:41	05:40:10
	Male	600	02:39:23	00:26:15	02:05:08	03:52:44
15–30	Female	3319	03:03:39	00:20:31	02:18:11	04:56:30
	Male	3800	02:28:39	00:15:20	02:01:39	03:51:37
**Minimum temperature range (°C)**						
0–8	Female	1537	02:59:40	00:18:37	02:19:19	05:40:10
	Male	1900	02:32:11	00:20:21	02:02:57	03:52:44
8–15	Female	2204	03:06:00	00:21:56	02:18:11	04:56:30
	Male	2500	02:28:32	00:15:02	02:01:39	03:51:37
**Atmospheric pressure range (mbar)**						
900–1013	Female	2514	03:04:26	00:21:48	02:19:12	05:40:10
	Male	3000	02:30:34	00:18:42	02:01:41	03:52:44
1013–1030	Female	1227	03:01:16	00:18:39	02:18:11	04:54:20
	Male	1400	02:29:08	00:15:01	02:01:39	03:20:31
**Precipitation range (mm)**						
0–10	Female	3629	03:03:20	00:20:46	02:18:11	05:40:10
	Male	4200	02:29:31	00:17:18	02:01:39	03:52:44
10–20	Female	12	03:52:22	00:40:34	02:40:38	04:54:20
	Male	100	02:57:40	00:15:25	02:16:20	03:19:25
20–50	Female	100	03:00:06	00:12:39	02:23:58	03:12:57
	Male	100	02:27:09	00:08:19	02:05:08	02:35:50

## Discussion

This study investigated the influence of environmental conditions such as temperature (i.e., mean temperature, daily highest, and lowest temperature), sunshine duration, precipitation, barometric pressure) on marathon race times in all editions of the Berlin Marathon since its first edition in 1974 until 2019 with the hypothesis to find an association between ambient temperatures and marathon race times as it has been reported for other large marathon races. The most important findings were: (1) overall runners became slower with increasing temperature and sunshine duration, (2) elite runners (i.e., top 3 and top 10) seemed to run faster and improved their race times when the temperature increased, where the top 10 women appeared to benefit more than the top 10 men, and (3) male top 100 runners seemed to benefit more than female top 100 runners from increasing temperatures.

### All Women and Men

A first important finding is that marathon race times showed a weak correlation with all weather variables for all women and men, however, only minimally significant with maximum temperature and sunshine duration, with the latter two variables strongly correlating with each other. In general, the masses (all runners) seemed to run slower and worsen their finish times with increasing temperature and sunshine duration. This finding confirms existing findings that marathon race times are impaired with increasing temperatures (Ely et al., 2007a,b, 2008; El Helou et al., 2012). For example, in the Boston Marathon, an increase by 1°C increased overall marathon race time from 00:01:47 h:min:s (Knechtle et al., 2019) to 00:01:53 h:min:s (Nikolaidis et al., 2019). Also, in the Stockholm Marathon, race times of slower marathoners were more affected by unfavorable weather conditions (Vihma, 2010).

An interpretation of the finding that slow runners were influenced by weather more than faster runners might be attributed to the fact that the slow runners spent more time exposed to environmental conditions (El Helou et al., 2012). It has been also supported that slow runners ran close to each other and this, in turn, might increase the heat stress (De Freitas et al., 1985). Moreover, it has been assumed that the ability to cope with increasing heat stress might be linked to physiological characteristics differing by performance level (Ely et al., 2007b).

### Elite (Top 3 and Top 10) Women and Men

A second important finding is that elite runners (i.e., top 3 and top 10) seemed to run faster when the temperature increased, with women also improving more than men in the top 10 sample. A potential explanation could be the nationality of elite marathoners that participate in large city marathons, as it is well-known that the fastest marathon runners originate from East Africa (i.e., Kenya and Ethiopia). This has been reported for the New York City Marathon (Vitti et al., 2020) and the World Marathon Majors (Boston, Berlin, Chicago, and New York) (Knechtle et al., 2017). A study investigating 1,174,331 finishers from the New York City Marathon showed that Ethiopians and Kenyans were the fastest and youngest in women and men, respectively (Vitti et al., 2020). Ethiopians and Kenyans are, however, also the fastest marathoners in the IAAF ranking (Nikolaidis et al., 2017). Most likely, East African runners are accustomed to competing in higher temperatures. However, their differences in body dimensions (Larsen and Sheel, 2015) and favorable somatotypical characteristics (Wilber and Pitsiladis, 2012) with lower body mass and lower body mass index (Larsen, 2003; Lucia et al., 2006; Mooses and Hackney, 2017) might also be of advantage in higher temperatures.

The discovery that elite women could profit from increasing temperatures for a fast marathon race time confirms the findings of Vihma (2010) investigating the Stockholm Marathon from 1980 to 2008 where effects of warm weather were less evident for female than for male runners. However, our results contradict the findings from Ely et al. (2008) investigating three Japanese women’s championship marathons. These authors found that increasing air temperatures slowed pace more in faster runners (i.e., winner, 25th place) than slower runners (i.e., 50th place, 100th place) although the temperatures were similar (cool 5–10°C and warm 15.1–21°C). Similarly, in an analysis of several marathon races, Ely et al. (2007b) reported a progressive decrease in marathon race times when temperature increased from 5 to 25°C for both women and men, where performance was more negatively affected for slower runners. These disparate findings might be explained by the fact that Berlin Marathon has the fastest marathon race course with the most marathon world records set^5^.

### Sub-Elite (Top 100) Women and Men

A third important finding was that the top 100 men improved their race times with increasing temperatures, but not the top 100 women. Most likely, this segment of runners also belongs to the elite section due to the large field of runners in the Berlin Marathon with 50,000–60,000 annual competitors)^6^. This discovery is in contrast to findings in a study on the Boston Marathon where all men competing between 1897 and 2018 were investigated (Nikolaidis et al., 2019). It has been reported that performance of all levels of groups decreased significantly when temperature increased. When temperature increased by 1°C, performance decreased by 00:01:53 h:min:s for all finishers, by 00:00:37 h:min:s in the top 100 finishers and by 00:00:38 h:min:s in the top 10 finishers (Nikolaidis et al., 2019). The disparate findings might be explained by the fact that the Boston Marathon was held and investigated for a considerably longer time frame (1897–2018) compared to the Berlin Marathon (1974–2019) and the Boston Marathon is a point-to-point race with ascents and descents, compared to the Berlin Marathon, which has a flat loop.

## Conclusion

In summary, in marathoners competing in the Berlin Marathon between 1974 and 2019, increasing temperatures and sunshine duration showed a different effect on different performance levels where most runners (i.e., the general mass of runners) became slower with increasing temperature and sunshine duration, but elite runners (i.e., top 3, top 10, and top 100) became faster with increasing temperatures, while sex differences exist. Elite women in the top 3 and top 10 seemed to profit more from increasing temperatures than elite top 100 women.

## Data Availability Statement

The raw data supporting the conclusions of this article will be made available by the authors, without undue reservation.

## Author Contributions

BK drafted the manuscript. DV performed the statistical analyses. EV collected all data. JA, VS, TR, and PN helped in drafting the manuscript. All authors approved the final version of the manuscript.

## Conflict of Interest

The authors declare that the research was conducted in the absence of any commercial or financial relationships that could be construed as a potential conflict of interest.

## Footnotes

1 www.bmw-berlin-marathon.com/impressionen/statistik-und-geschichte/ergebnisarchiv/

2 www.dwd.de/DE/leistungen/klimadatendeutschland/klarchivtagmonat.html? nn=16102

3 https://www.python.org/

4 https://colab.research.google.com/

5 www.runnersworld.com/races-places/a20823734/these-are-the-worlds-fastest-marathoners-and-marathon-courses/

6 www.bmw-berlin-marathon.com/en/impressions/statistics-and-history/numbers-and-records/
